# FISH comets show that the salvage enzyme TK1 contributes to gene-specific DNA repair

**DOI:** 10.3389/fgene.2014.00233

**Published:** 2014-08-08

**Authors:** Katherine A. McAllister, Akeel A. Yasseen, George McKerr, C. S. Downes, Valerie J. McKelvey-Martin

**Affiliations:** ^1^School of Biomedical Sciences, University of UlsterColeraine, UK; ^2^Department of Pathology and Forensic Medicine, Faculty of Medicine, University of KufaKufa, Iraq

**Keywords:** FISH comet, gene-specific repair, radiation damage, thymidine kinase

## Abstract

Thymidine kinase 1 (TK1) is a salvage enzyme that phosphorylates thymidine, imported from surrounding fluids, to create dTMP, which is further phosphorylated to the DNA precursor dTTP. TK1 deficiency has for a long time been known to cause increased cellular sensitivity to DNA damage. We have examined preferential strand break repair of DNA domains in TK1^+^ and TK1^-^ clones of the Raji cell line, by the Comet-FISH technique, in bulk DNA and in the actively transcribed tumor suppressor (TP53) and human telomerase reverse transcriptase (hTERT) gene regions, over 1 h after 5Gy γ-irradiation. Results showed that repair of the TP53 and hTERT gene regions was more efficient in TK1^+^ compared to TK1^-^ cells, a trend also reflected to a lesser degree in genomic DNA repair between the cell-lines. The targeted gene-specific repair in TK^+^ cells occurred rapidly, mainly over the first 15 min repair-period. Therefore, TK1 is needed for preferential repair of actively transcribed regions, through a previously unsuspected mechanism. In principle, TK1 could exert its protective effects through supply of a supplementary dTTP pool for accurate repair of damaged genes; but Raji TK1^+^ cells in thymidine free media still show preferential repair of transcribed regions. TK1 therefore does not exert its protective effects through dTTP pools, but through another unidentified mechanism, which affects sensitivity to and mutagenicity by DNA damaging agents.

## INTRODUCTION

Human thymidine kinase 1 (TK1) is a salvage enzyme that phosphorylates thymidine to create dTMP, which is later converted by thymidylate kinase and nucleoside diphosphate kinase to dTTP, a precursor for DNA metabolism (Segura-Pena et al., 2007). This is usually the minor pathway for dTTP synthesis, subsidiary to the *de novo* pathway in which ribonucleotide reductase converts UDP to dUDP, which is dephosphorylated to dUMP and then methylated by thymidylate synthase to dTMP. TK1 is not essential for viability (Dobrovolsky et al., 2003). A related enzyme, TK2, is mitochondrial and irrelevant to nuclear events.

While the functions of TK1 are clearly related to the processes of DNA replication and cell proliferation, many studies in the literature demonstrate a protective role for the protein during cellular responses to DNA damage. Human and rodent *in vitro* studies show that deficiency of the protein causes increased sensitivity to a diverse range of DNA damaging agents, including ionizing radiation (McKenna and Hickey, 1981; McKenna and Yasseen, 1982; McKenna et al., 1988; al-Nabulsi et al., 1994; Best et al., 1994; Wakazono et al., 1996). In this context, it is noticeable that TK1 mRNA is induced not only in S phase and G2 but also following ionizing radiation, causing a concomitant increase of enzymatic activity (Boothman et al., 1994; Wei et al., 1999; Castro Kreder et al., 2002; Haveman et al., 2006). More recently, it has been shown that TK1 is upregulated in different tumor types in response to DNA damage, and that the cellular response to genotoxins causes nuclear localization of TK1; an interesting finding given that the salvage enzyme has previously been regarded as solely cytoplasmic (Chen et al., 2010). These studies suggest that TK1 may somehow affect DNA metabolism in a way not obviously explained by its salvage role.

The protective effect of TK1 might be due to its maintaining of the efficiency of DNA repair during recovery from genotoxic insults. TK1 deficiency not only restricts the dTTP pool but upsets the balance of all four dNTP precursors (Wilkinson and McKenna, 1989). Deficiency of the TK1 regulated dTTP pool causes decreased viability and increased mutation after treatment with mutagenic agents (Wakazono et al., 1996; Kubota et al., 1998; Hyland et al., 2000). Analagously, a reduction in dTTP pools by silencing of thymidylate kinase also sensitizes cells to DNA damage (Hu and Chang, 2008). Recent work in colon carcinoma cells (Chen et al., 2010) showed that knockdown of TK1 decreases the efficiency of double-strand DNA break repair during recovery from DNA damage. Chen et al. (2010) also found that the TP53 status of the tumor cells affected the level of TK1 after DNA damage.

We address in this study TK1’s effect on the kinetics of repair after DNA damage, in particular the repair of DNA damage occurring in specific areas of the genome. Many studies have documented that repair occurs at a more accelerated rate in transcribed gene regions such as TP53, compared to that of total DNA (reviewed by Sarasin and Stary, 2007). Such transcription-coupled repair has the capacity to reduce mutations in vital domains of the genome. While earlier DNA repair studies using murine cells have shown that TK1 deficiency does not prevent bulk excision repair from occurring after genotoxic insult (McKenna and McKelvey, 1986), there has been no evidence for its effect on the faster repair that occurs with damage to specific gene regions. The Comet assay is an ideal method for investigations of the kinetics of repair of damage induced in nuclear DNA by ionizing radiation. The strand breaks induced are typically rejoined quickly, most breaks disappearing within 30 min (reviewed by Frankenberg-Schwager, 1989). The method when combined with the use of fluorescent hybridization probes (Comet-FISH) allows the study of repair kinetics in gene-regions of interest by quantifying the rate of strand break repair within such target genes (Santos et al., 1997; McKelvey-Martin et al., 1998; McKenna et al., 2003; Horvathova et al., 2004; Glei et al., 2007).

This investigation was carried out to determine whether TK1 may affect the damage response in two selected transcribed gene regions [TP53 and human telomerase reverse transcriptase (hTERT)] both of which are actively transcribed by Raji TK1^+^ and TK1^-^ clones, and thereby to provide further insight into potential mechanisms of mutagenesis and carcinogenesis induced by misrepair of DNA. Human TP53 is a well-characterized tumor suppressor gene and is located on the short arm of chromosome 17 (17p13.1; Matlashewski et al., 1984). TP53 is induced by γ-irradiation (McKay et al., 1999) and is in a domain seen to be rapidly repaired after γ-irradiation in comparison to other genes (McKenna et al., 2003, 2012). The hTERT gene (5q15.33), which codes for hTERT, the catalytic subunit of the telomerase enzyme, is upregulated in the majority of cancer cells (Hiyama and Hiyama, 2003); strand break repair of γ-irradiation damage to the hTERT gene domain is likewise rapid (McKenna et al., 2012).

The position of FISH signals within the Comet head or tail indicates whether or not damage has occurred, or not yet been repaired, to the gene-region selected by the probe; this can be compared to damage in global DNA. In this study we applied the Comet-FISH protocol to study DNA repair in TK1^+^ and TK1^-^ clones of the human lymphoblastoid cell line Raji. The possibility that TK1 exerts its protective effects through the dTTP pool was also investigated by growing Raji TK1^+^ cells in thymidine free media. Repair was followed over a 1-h period following exposure to 5Gy γ-irradiation.

## MATERIALS AND METHODS

### CELL-LINES AND CELL CULTURE

TK1^-^ and TK1^+^ clones of the Raji lymphoblastoid cell line were grown in RPMI 1640 culture media, supplemented with 15% fetal bovine serum (FBS), 2 mM glutamine, and antibiotics (100 U/ml penicillin, 0.1 mg/ml streptomycin)_._ Raji cell-lines were purchased from the European Collection of Cell Cultures (ECACC, Salisbury, UK). In thymidine free experiments, TK1^+^ cells were first washed in PBS, then cultured in thymidine free media containing dialyzed FBS to remove the salvage dTTP pool. The Werner syndrome (WS) cell line was obtained from the Coriell Cell Repository (Camden, NJ, USA) and maintained in minimum essential medium, supplemented with 15% FBS and glutamine, penicillin, and streptomycin as above. The GM38 normal human fibroblast cell-line was obtained from the Human Genetic Msitory (Camden, NJ, USA) and cultured in Eagle’s minimum essential medium supplemented with 20% FBS, 4% essential amino acids, 2% non-essential and glutamine, penicillin, and streptomycin as above. The GM38 cell line actively expresses TP53 (Glei et al., 2007) and served both as a positive and negative PCR control to study TP53 and hTERT gene expression, respectively. The WS cell line is immortalized with the hTERT gene and therefore served as a PCR positive control to examine hTERT gene expression in Raji cells.

### CHARACTERIZATION OF RAJI CELLS

Raji TK1^-^ cells were cultured in 5 μg/ml trifluorothymidine (TFT; Sigma, Poole, UK), which is lethal to TK1^+^ cells, to confirm the cellular phenotype. TK assays were used to determine the activity of TK1 in Raji cells (McKenna et al., 1985). Metaphase spreads (*n* = 100) were examined to quantify chromosome numbers in Raji cells for general characterization and to correlate with later FISH spot numbers (McKenna et al., 2003).

### hTERT AND TP53 GENE EXPRESSION

Total RNA was extracted from each of the four cell lines using an RNeasy Mini kit (Qiagen, Mississauga, MD, USA); integrity was verified by 1% gel electrophoresis and quantity and quality determined using a NanoDrop ND-1000 UV-VIS Spectrophotometer (Wilmington, DE, USA). Complementary DNA was generated using a Superscript II RNase H-Reverse Transcriptase Kit (Invitrogen, Renfrew, UK) according to the manufacturer’s protocol. β-actin served as the internal control in RTPCR reactions. Template cDNA was amplified for PCR using GoTaq DNA Polymerase (Promega, Southampthon, UK) in the presence of primers specific for the β-actin gene: 5′AGAAAATCTGGCACCACACC-3′ (sense) and 5′CCATCTCTTGCTCGAAGTCC-3′ (anti-sense), or primers specific for the hTERT gene: 5′CTCACCTTCAACCGCGG-3′ (sense) and 5′TTGCTGAAATGGGAGCT-3′ (anti-sense). Reaction conditions for β-actin were 40 cycles of denaturation at 96°C for 1 min, annealing at 55°C for 30 s and extension at 72°C for 10 min; conditions for hTERT were 35 cycles of denaturation at 94°C for 45 s, annealing at 60°C for 45 s, and extension at 72°C for 90 s. Platinum TAQ DNA polymerase (Invitrogen, Renfrew, UK) was used to amplify the TP53 gene using the following primers: 5′-TCACTGCCATGGAGGAG-3′ (sense) and 5′-TCAGTGGGGAACAAGAAG-3′ (anti-sense). PCR reactions conditions were: 35 cycles of denaturation at 94°C for 1 min, annealing at 50°C for 30 s, and extension at 72°C for 2 min. A final elongation step of 10 min was used to ensure that any remaining single stranded DNA was completely copied.

### COMET-FISH ASSAY

For the alkaline Comet assay, cells were embedded in agarose onto Dakin fully frosted microscopic slides (Labcraft, London, UK), and subjected to a dose of 5Gy irradiation using a Cs^137^ source. Following irradiation slides were quickly immersed in repair medium at 37°C for either 15, 30, or 60 min, then drained and placed in alkaline lysis solution (2.5 M NaCl, 100 mM Na_2_EDTA, 10 mM Tris, pH 10, and 1% Triton X-100 added before use) for 1 h at 4°C. DNA was left to unwind in electrophoresis buffer (300 mM NaOH, 1 mM Na_2_EDTA, pH13) for 20 min. Electrophoresis was conducted for 20 min at 25 V and 300 mA, after which slides were washed with three changes of neutralization buffer (0.4 M Tris, pH 7.5).

Target DNA was prepared for FISH by a 5 min wash with 2X SSC (3 M sodium citrate, pH 5.3), and subsequent dehydration with increasing concentrations of ethanol (70, 85, 100%). Multicolor Comet-FISH was performed using a fragmented locus-specific identifier (LSI) Spectrum-Orange-labeled TP53 DNA probe spanning a 140 kb region containing the 20 kb TP53 gene (Vysis, Surrey, UK), in conjunction with a LSI direct labeled Spectrum Green hTERT probe, spanning a 180 kb region containing the 40 kb hTERT gene (Q-Biogene, Cambridge, UK). Equal concentrations of a TP53 and hTERT probe mix were applied to each slide. Co-denaturation of DNA was performed at 80°C (2 min) followed by a 16-h hybridization period at 37°C in a dark, humidified chamber. Following hybridization, excess probe was removed at 45°C using three 10 min washes of 50% formamide and 2X SSC, a 10 m wash with 2X SSC and a final 5 min 2X SSC and 0.1% Igepal wash. Slides were left to air dry for 1 h then counterstained with 16 μl 4′6-diamidino-2-2phenylindole (DAPI) for immediate observation. All reagents for the Comet-FISH assay were purchased from Sigma, Poole, UK unless otherwise stated.

### COMET FISH MICROSCOPY AND ANALYSIS

The slides were viewed using an epifluorescence microscope (Nikon Eclipse E400) using a triple bandpass filter (Chroma HiQ) that enabled the simultaneous detection of DAPI (overall genome) and both spectrum orange (TP53 gene region) and spectrum green (hTERT gene region) for the enumeration of FISH signals. Standard Comet parameter measurements, including % comet tail DNA, were recorded using the Komet 5.0 digital imaging system (Kinetic Imaging Ltd., Liverpool, UK).

Studies of repair of bulk DNA selected the measurement % comet tail DNA, as the amount of DNA in the tail (relative to the comet head) is proportional to the number of γ-irradiation induced strand breaks. For DNA damage and repair to the p53 and hTERT gene regions the numbers of fluorescent probes detected in the comet head and tail were quantified over timed intervals during a 1-h incubation period. The repositioning of the gene-specific signals from the comet tail into the head over the incubation period provides evidence for repair of lesions occurring within and around the TP53 and/or hTERT gene locus. The level of DNA repair in the TP53 and hTERT gene region was examined using the parameter % TP53/hTERT signals in the comet tail.

### STATISTICAL ANALYSIS

One slide was analyzed from each dose point, and per slide 50 comets were scored. Three independent experiments were conducted to generate each data point (150 cells scored in total per cell line). The normality distribution of the entire comet-FISH dataset was visually inspected from normal probability plots and evaluated using the Shapiro–Wilk *W*-test. The Mann–Whitney test was applied to evaluate comparisons between the non-parametric comet-FISH dataset for the TK1^+^ and TK1^-^ clones. A difference with a *p* < 0.05 was deemed significant. Results were analyzed using GraphPad Prism version 5 (UK).

## RESULTS

### CHARACTERIZATION OF RAJI CELLS

**FIGURE 1:**
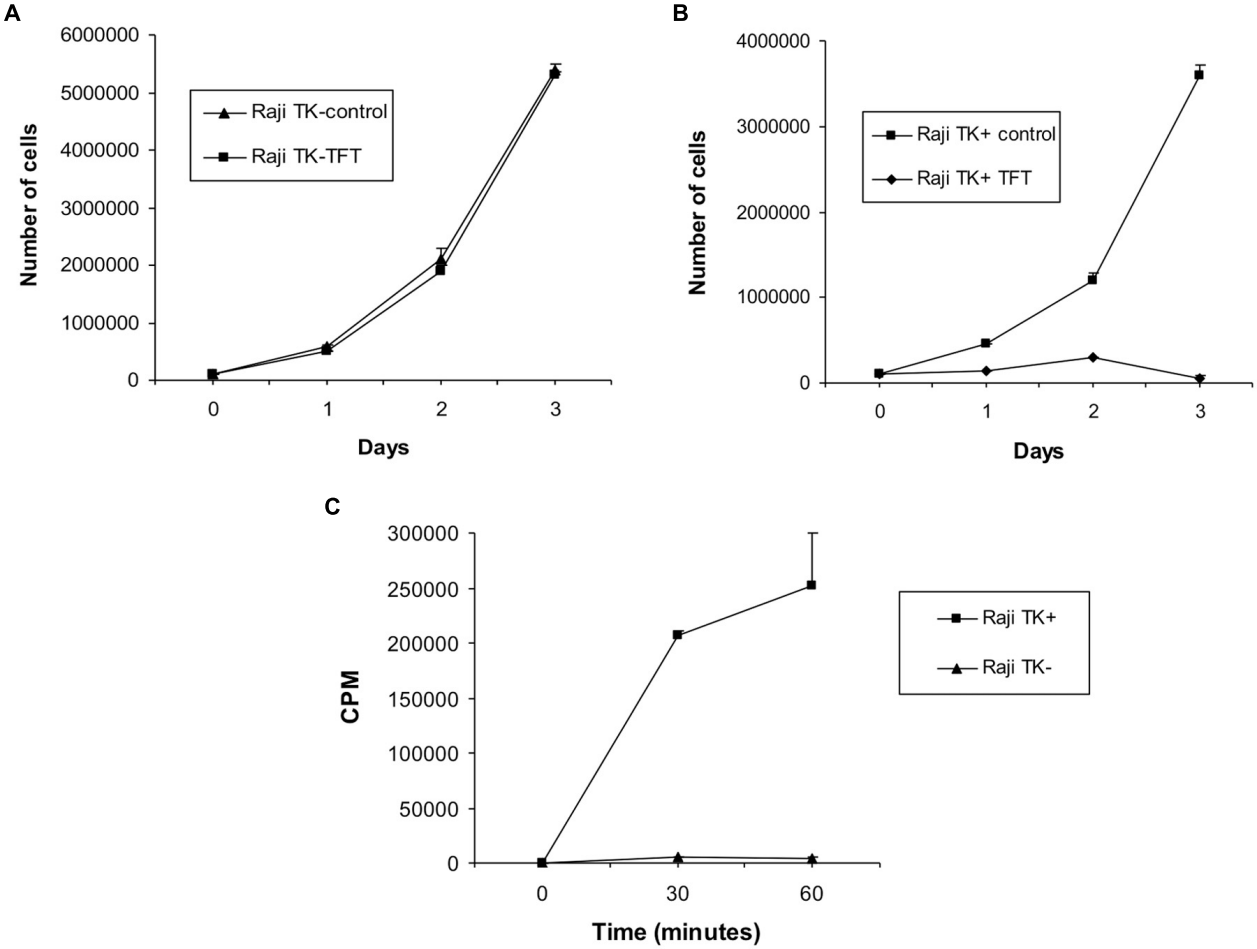
**Confirmation of cellular phenotype in Raji cells. (A)** Effect of culturing TK1^-^ cells and **(B)** TK1^+^ cells in 5 μg/ml TFT compared to untreated control cells. Culture of Raji cells in TFT proved toxic to the TK1^+^ clones whereas TK1^-^ cells were resistant. Only cells containing functional TK1 can incorporate TFT and uptake of the chemical results in cell death. Therefore Raji TK1^-^ cells lack TK1 functional protein. **(C)** Tritium counts obtained from Raji TK1^+^ and TK1^-^ clones during the TK assay expressed in counts per minute (CPM). The graph shows that the incorporation of ^3^H-thymidine is greatly reduced in Raji TK1^-^ cells compared to TK1^+^. The ability of cells to incorporate ^3^H-thymidine into nucleic acids is an approximate measure of cellular thymidine kinase activity; therefore Raji TK1^-^ cells lack functional TK1 protein. The results in each graph data-point represent the mean ± SEM of three independent experiments.

The Raji cells used are from lines that were originally created in the 1990s: we have therefore checked that they have retained their original phenotypes. The TK1^+^ line retain sensitivity to the toxic thymidine analog trifluorothymidine (Karran et al., 1990), while TK1^-^ cells which cannot metabolize it remain resistant. Correspondingly, incorporation of tritiated thymidine is linear in TK1^+^ cells, negligible in TK1^-^ (**Figure 1**). The TK1^-^ nature of the Raji clones, first established several years ago (Hampar et al., 1971), has therefore been maintained.

Metaphase spread analysis revealed that Raji TK1^+^ and TK1^-^ cells have a near diploid mainline featuring a population of cells ranging from 42–50 chromosomes. The modal chromosome number for TK1^-^ was 46 while for TK1^+^ it was 47. A small, more aneuploid population in both cell-lines increased the overall mean number of chromosomes to 54 and 52 for TK1^-^ and TK1^+^ respectively.

**FIGURE 2:**
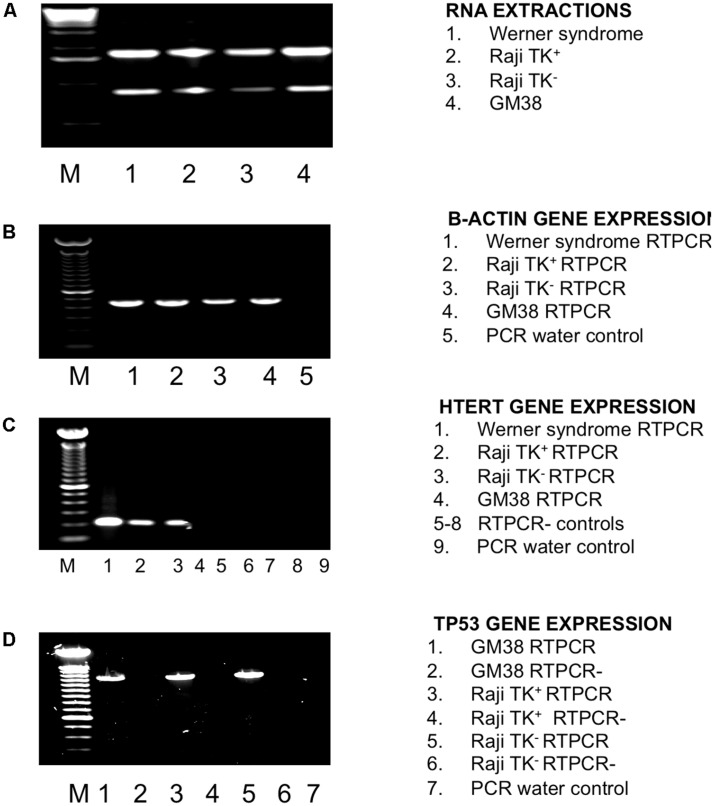
**Investigation of TP53 and hTERT status in Raji cells using RTPCR. (A)** RNA extraction. Lane 1 Werner Syndrome (WS), lane 2 TK1^+^, lane 3 TK1^-^, lane 4 GM38, M is a 1 kb ladder. **(B)** β-actin gene expression internal control check as detected by a 435 amplicon in all cell-lines. Lane 1 WS, lane 2 TK1^+^, lane 3 TK1^-^, lane 4 GM38 and lane 5 PCR water control. M is a 100 bp ladder. **(C)** HTERT gene expression detected by a 200 bp amplicon. HTERT gene expression was found in the WS cell-line, TK1^+^ and TK1^-^ cells, but not GM38. Lane 1 WS, lane 2 TK1^+^, lane 3 Raji TK1^-^, lane 4 GM38 lane 5 –RTPCR control (-RT) WS, lane 6 –RT TK1^+^, lane 7 –RT TK1^-^, lane 8 –RT GM38, lane 9 PCR water control. M is a 100 bp ladder **(D)** TP53 gene expression as detected by a 1220 bp band present in all cells. Lane 1 GM38, lane 2 GM38 –RT, lane 3 Raji TK1^+^, lane 4 Raji TK1^+^ –RT, lane 5 Raji TK1^-^, lane 6 Raji TK1^-^ –RT, lane 7 PCR water control. M is a 100 bp ladder.

We have used TP53 and hTERT as markers for transcribed gene repair: it was therefore necessary to check they are in fact transcribed in both cell lines. They are, as shown in (**Figure 2**). A sample was considered positive for the hTERT gene expression by the presence of a 200 base pair amplicon (**Figure 2C**), as was the case for every cell-line except the fibroblast cell-line GM38. TP53 gene expression was also detected in both Raji clones and the normal fibroblast, GM38, as shown in **Figure 2D** by the presence of a 1220-base pair amplicon. Sequencing data of the TP53 gene in the Raji cell-lines also confirmed that the alleles were identical in both, and therefore any difference in DNA repair could not be accounted for by mutations in this key DNA repair gene (sequencing data not shown, supplementary).

### DNA REPAIR IN TK1^+^ AND TK1^-^ CELLS

**FIGURE 3:**
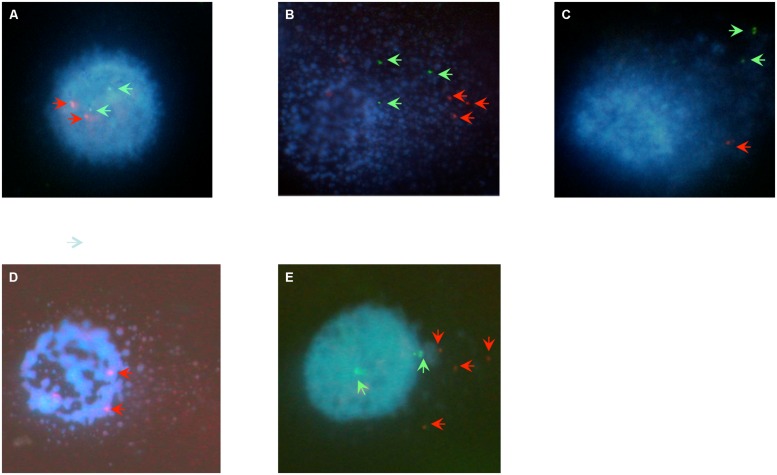
**Digital imaging of comet-FISH cells.** The position of TP53 and hTERT hybridization spots in the comet head or tail specifies whether it lies in, or close to, a region of intact or damaged DNA. Observations for imaging were made at a final magnification of ×600 (Nikon ×60 Fluor lens). **(A)** An untreated Raji cells displays minimal DNA damage as evidenced by the intact comet head and minimal comet tail. Two TP53 (red arrows) and two hTERT (green arrows) hybridization spots are visible in the intact comet head. **(B,C)** Immediately following 5Gy γ-irradaition a large comet tail is visualized, indicating a large amount of overall DNA damage, with red TP53 and green hTERT hybridization spots in the comet tail indicating that radiation-induced strand breaks have occurred within or close to the vivinity of both genes of **(B)** TK^+^ and **(C)** TK^-^ cells. **(D)**. At 15 min of repair the TK^+^ cells have repaired most gene specific damage as depicted here for the TP53 gene region, as evidenced here by the FISH spots being located in the head. **(E)** Raji TK^-^ cells at 60 min post-irradiation typically feature TP53 and/or HTERT FISH spots located in the comet-head as gene repair has begun to recover. However, FISH tail spots still remain in higher numbers than TK^+^, this picture showing an example of damage remaining in the TP53 locci of TK^-^ cells at 60 min.

Comet-FISH experiments were evaluated by quantifying the number of TP53 and hTERT hybridization spots located in the comet head or tail of TK1^+^ and TK1^-^ at each repair incubation time as shown both in representative images in **Figure 3** and the data in **Table 1**. FISH comet tail spots were almost entirely absent in both un-irradiated controls; and on account of the aneuploidy and the post-replicative elements in the population, the average spot number in the comet head was above two (**Figure 3A**). Post-irradiation, both cell-lines showed an increase in TP53 and hTERT probe signal in the comet tail, which is indicative of damage to both gene regions (**Figures 3B,C**). A notable finding in Raji TK1^+^ cells (**Table 1**) was a rapid rate of gene-specific repair that featured significant decreases in the number of TP53 and hTERT tail spots at 15 min compared to TK1^-^ (*p* < 0.0001) and also at 30–60 min (*p* < 0.0001). Repair continued at a slower rate for the remainder of the repair period in TK^+^ cells so that by 60 min there was no signal left of either probe in the tail (**Figure 3D**). In comparison, gene-specific repair was found to be stalled in TK1^-^ cells and the comet-tail FISH signals for each gene probe began to diminish only after 15 min (**Figure 3E**).

**Table 1:** Data generated by the comet-FISH assay for both Raji TK^+^ and TK^-^cells at 0, 15, 30, and 60 min following a dose of 5Gy γ-irradiation.

		Repair time-point (minutes)			
Cell-line	Percentage (%)	0	15	30	60
Raji TK^+^	DNA in the comet tail	31.76 ± 1.25	18.36 ± 1.24	12.82 ± 1.41	8.05 ± 0.74
	TP53 comet tail signals	70.05 ± 2.20	22.31 ± 2.61	18.22 ± 3.03	7.53 ± 2.19
	hTERT comet tail signals	77.50 ± 1.89	29.77 ± 3.12	15.03 ± 2.37	6.82 ± 1.88
Raji TK^-^	DNA in the comet tail	30.53 ± 0.99	22.98 ± 1.46	15.82 ± 1.46	10.98 ± 0.79
	TP53 comet tail signals	62.28 ± 2.45	57.49 ± 2.41	37.63 ± 3.46	17.12 ± 2.36
	hTERT comet tail signals	74.05 ± 3.16	59.59 ± 3.02	39.87± 3.80	29.82±3.19

*Each data point represents the mean value obtained from three independent experiments; 50 cells were quantified per replicate. Comet-FISH experiments were first evaluated by quantifying the number of TP53 and hTERT hybridization spots located in the comet head or tail of TK1^+^ and TK1^-^ at each repair incubation time.*

**FIGURE 4:**
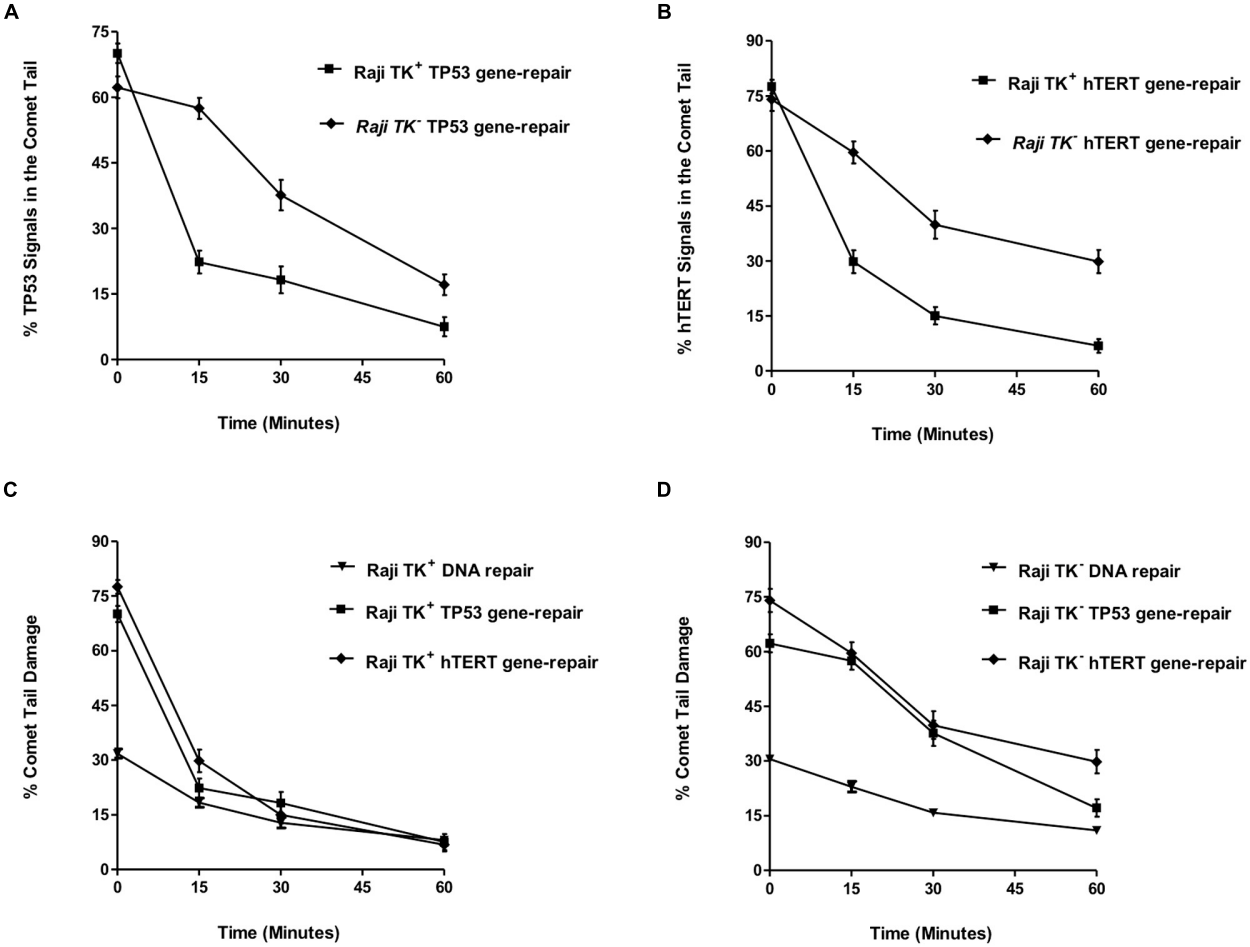
**TP53 and hTERT gene-repair in TK1^-^ and TK1^+^ cells and comparison with the overall genome. (A)** shows the % TP53 tail signals and **(B)** the % hTERT tail signals over the repair time period after 5 Gy-irradiation. **(C,D)** compares DNA repair between the overall genome and specific gene-regions. These findings demonstrate that preferential repair is occurring in the TP53 and hTERT domains of Raji TK1^+^ but not in TK1^-^ cells. In each graph, fifty cells were analyzed at each time-point for each experimental replicate. Each data point represents the mean ± SEM of three independent replicate experiments.

The level of strand break repair in the specific gene-regions was compared between TK1^+^ and TK1^-^ cells using the parameter “% TP53/hTERT signals in the comet tail” (**Figures 4A,B**). There was found to be a significant reduction (*p* < 0.0001) in the % of TP53 and hTERT tail signals at each time-point in Raji TK1^+^ cells compared to TK1^-^ (**Figures 4A,B**). The most prominent occurred at 15 min, with a difference between the Raji clones of 35.18 ± 3.56 for TP53 tail spots and 29.82 ± 4.34 for hTERT tail spots. The delayed reduction of % FISH signals in the comet tail for each gene over the hourly incubation period demonstrates that TK1^-^ are severely compromised in gene region repair kinetics compared to TK1^+^.

This may be compared to the levels of radiation-induced strand breaks in bulk genomic DNA, which were reduced over the 1-h repair period in a similar pattern for both Raji TK1^+^ and TK1^-^ cells. The percentage of total DNA in the Comet tail ranged from 32 to 8 % in TK^+^ cells and from 31 to 10.11% in TK^-^ cells (**Figures 4C,D**). Statistics showed that TK^+^ cells had significantly but slightly greater levels of bulk DNA repair at both 15 and 30 repair minutes (*p* > 0.05) compared to TK1^-^ cells.

The level of strand break repair in the specific gene-regions was compared between TK1^+^ and TK1^-^ cells using the parameter “% TP53/hTERT signals in the comet tail” (**Figures 4A,B**). There was found to be a significant reduction (*p* < 0.0001) in the % of TP53 and hTERT tail signals at each time-point in Raji TK1^+^ cells compared to TK1^-^ (**Figures 4A,B**). The most prominent reduction occurred at 15 min, with a difference between the Raji clones of 35.18 ± 3.56 for TP53 tail spots and 29.82 ± 4.34 for hTERT tail spots. The delayed reduction of % FISH signals in the comet tail for each gene over the hourly incubation period demonstrates that TK1^-^ are severely compromised in gene region repair kinetics compared to TK1^+^.

**Figure 4C** evaluates the mean % comet tail damage in Raji TK1^+^ cells and shows an accelerated reduction of tail TP53 and hTERT spots compared to tail bulk DNA. **Figure 4D** demonstrates how reduction of % comet tail DNA damage occurs at more similar rates for both bulk DNA and the TP53 and hTERT gene regions in the TK1^-^ cell-line. The pattern of repair observed in each gene region would suggest that preferential repair is occurring in the TP53 and hTERT domains of Raji TK1^+^ but not in TK1^-^ cells.

### EFFECT OF THYMIDINE FREE CULTURE ON PREFERENTIAL REPAIR CAPABILITIES OF RAJI TK1^+^ CELLS

**FIGURE 5:**
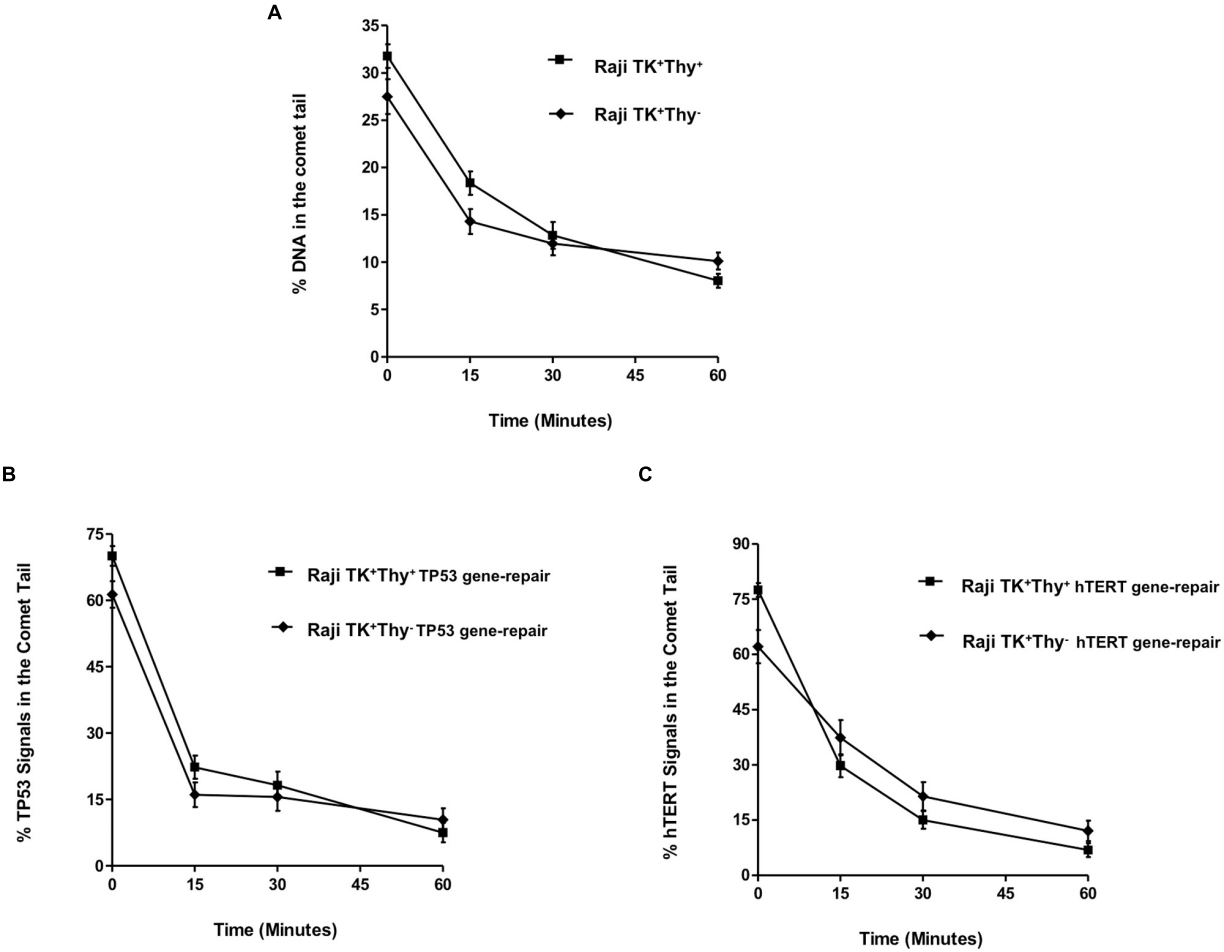
**Effect of thymidine salvage depletion in Raji TK1^+^ cells. (A)** depicts the repair of bulk DNA, while graphs **(B,C)** show the % TP53 and hTERT tail signals over the repair time period after 5 Gy-irradiation. The rapid gene repair observed clearly demonstrates that culturing of TK1^+^ cells in thymidine free media has no effect on the level of preferential repair. Fifty cells were analyzed at each time-point for each experimental replicate. Each data point represents the mean ± SEM of three independent replicate experiments.

The growth of TK1^+^ cells in thymidine free media (Raji TK^+^ Thy^-^) had no impact on the proficiency of single strand repair of bulk DNA in Raji TK1^+^ cells (**Figure 5A**) or gene region-specific repair of TP53 (**Figure 5B**) or hTERT (**Figure 5C**). **Figure 5B** shows TP53 repair in Raji TK1^+^ Thy^+^ and Raji TK1^+^ Thy^-^ cells. Statistical analysis found no significant difference in the % of TP53 tail signals between TK1^+^ Thy^+^ and Raji TK1^+^ Thy^-^ cells (*p* > 0.05). Statistical analysis likewise found no significant difference in the % hTERT tail signals at any given time points. These experiments show that culturing of TK1^+^ cells in thymidine free media has no effect on the level of preferential repair.

## DISCUSSION

The misrepair of DNA damage can result in tumorigenesis through mutational activation of proto-oncogenes and inactivation of tumor suppressor genes, causing a variety of human cancer syndromes. Although TK1 deficiency is known to cause a clear increase in DNA damage sensitivity and mutagenicity, and the likelihood of increased carcinogenesis, the DNA repair process on which the salvage enzyme exerts its protective effects remains to be elucidated. While our results in human cells show that TK1 deficiency can reduce the rate of bulk DNA repair, the most prominent finding is the clear role for TK1 in repair of damage occurring in specific gene regions. It is of further interest to monitor DNA repair processes in actual cancer cell-lines such as Burkitt’s lymphoma-derived Raji, as a full appreciation of the mechanisms that govern DNA repair efficiency in such cells could help lead to the development of novel chemotherapeutic agents, and also help predict patient response to radiotherapy (McKenna et al., 2008).

We confirmed that TP53 and hTERT are substrates for preferential repair of gene sequences repair in Raji cells – intrinsically probable, since TP53 is transcribed throughout the cell cycle (Liu and Chen, 2006) and further induced by DNA-damaging agents, while the hTERT gene is upregulated in malignant cells such as Raji (Ducrest et al., 2002). RTPCR analysis found that both TK1^+^ and TK1^-^ clones actively expressed the hTERT and TP53 genes. Also, since the correct function of the TP53 gene product is a major factor in mediating DNA repair processes (reviewed by McKay et al., 1999) it is possible that disparity in DNA repair between TK1^+^ and TK1^-^ cells could arise if there were different TP53 mutations in either cell-line. Sequencing results for both TK1^+^ and TK1^-^ clones confirmed that the TK1^+^ and TK1^-^ clones have the same TP53 mutations and should therefore have the same tumor suppressor protein function during DNA repair.

The Comet-FISH data show that although TK deficiency does not prevent the repair of strand breaks in bulk DNA, it is slower in the TK1 deficient cell-line. The slight deceleration in bulk DNA repair in TK1^-^ is likely a reflection of the markedly reduced repair occurring in damaged regions containing transcribed genes. Our data shows that Raji TK1^-^ cells display poor initial repair of regions containing transcribed genes (as evidenced by a slower reduction of TP53 and hTERT tail hybridization signals). The rapid TP53 gene region repair demonstrated in TK1^+^ cells is also consistent with other studies of repair in this gene (McKenna et al., 2003; Horvathova et al., 2004). These results clearly demonstrate that TK1 is necessary for the rapid repair of both the TP53 and hTERT gene region.

One possibility to explain the difference in preferential repair between TK1^+^ and TK1^-^ clones is that TK1 provides a dTTP pool that facilitates the rapid repair of damaged genes. Raji TK1^+^ cells were therefore cultured in thymidine free media to deprive TK of its substrate, and damage induced gene region specific and bulk DNA repair were assessed. The results showed that the preferential repair capabilities of TK1^+^ cells were unaffected: therefore TK1 does not exert its effects through deoxyribonucleotide pools, otherwise repair of TK1^+^ cells would have been rendered similar to that of repair deficient TK1^-^ cells.

We have thus demonstrated that TK1 is a novel factor regulating preferential, gene-specific repair, operating not through its enzymic role but by some other mechanism: possibly activation of another protein involved in the repair process. There is no current evidence for direct interaction of TK1 protein with any known component of the single-strand break repair pathway. If one were to speculate, one might propose as a possible candidate for direct or indirect dependence on TK protein the DNA 3′phosphatase, in some circumstances a rate-limiting factor in single-strand break repair (Breslin and Caldecott, 2009). We note that defects in TK are well established as being mutagenic, and hence they are presumably also carcinogenic.

## Conflict of Interest Statement

The authors declare that the research was conducted in the absence of any commercial or financial relationships that could be construed as a potential conflict of interest.
